# Anatomical study of the medial sural artery perforator flap and its application in the reconstruction of infected defects

**DOI:** 10.3389/fcimb.2026.1907707

**Published:** 2026-08-03

**Authors:** Lijun Zhang, Ping Peng, Zhonggen Dong, Jianwei Wei, Shu Cao

**Affiliations:** 1 Department of Orthopedics, The Third Xiangya Hospital, Central South University, Changsha, Hunan, China; 2 Department of Orthopedics, The Second Xiangya Hospital, Central South University, Changsha, Hunan, China; 3 Department of Orthopedics, Hunan Provincial People’s Hospital, The First Affiliated Hospital of Hunan Normal University, Changsha, Hunan, China

**Keywords:** anatomical study, choke vessels, clinical application, infected defects, medial sural perforator flap

## Abstract

The medial sural artery perforator (MSAP) flap is widely used clinically for infected soft tissue defect repair and reconstruction due to its numerous advantages. However, the safe boundary of it was limited. The aim of this study was to explore the causes underlying the limited boundary of the MSAP flap via an anatomical study and to present the results of our clinical utilization of this flap for the reconstruction of infected defects. Five fresh lower limb specimens were perfused and then radiographed. From April 2023 to July 2025, 7 MSAP flaps were elevated in 7 patients for the reconstruction of infected soft-tissue defects over the knee, middle and distal leg, and heel. The reconstruction outcomes were documented and evaluated. The arteriography revealed that the anastomoses between adjacent medial sural artery perforators, as well as those between medial sural artery perforators and surrounding perforators, were choke anastomoses. All of the 7 flaps survived completely without any indications of arterial insufficiency or venous congestion. No clinical signs of persistent or recurrent infection, wound dehiscence, or flap necrosis were detected during the available follow - up period. These findings demonstrate that the MSAP flap can be effectively utilized to repair small - and medium - sized infected defects on the extremities. The possible factors influencing the limited harvestable boundary of this flap are that all adjacent perforators are anastomosed through choke vessels.

## Introduction

Infectious skin and soft-tissue defects resulting from trauma or chronic osteomyelitis are clinically prevalent, and their management poses numerous challenges (Guiotto et al., 2026; Taha et al., 2026; Zheng et al., 2016). Flap transplantation is one of the primary surgical methods for reconstructing this type of defect (S. Cao et al., 2025; Peng et al., 2022a; Tao et al., 2024). The flap can resurface the defect and restore the local barrier function, thereby reducing the invasion of external bacteria. The flap’s abundant blood supply not only facilitates the delivery of anti-infective mediators, such as antibiotics and cytokines, to the infected area but also promotes the absorption and removal of infected and necrotic tissues, which contributes to the control of the infection.

The medial sural artery perforator (MSAP) flap boasts numerous advantages, including a reliable blood supply and an appropriate thickness. Consequently, it is extensively utilized clinically for the repair of skin and soft tissue defects in the extremities as well as the oral and maxillofacial regions (Dattilo et al., 2025; Mohamed et al., 2024). With the advancement of surgical techniques and the in-depth research on anatomy, various technical forms, such as chimeric flaps and lobed flaps, have gradually emerged (S. Cao et al., 2025; Fritz et al., 2024; Saleh Mohamed et al., 2025). The scope of application has been gradually broadened, and the survival rate of flaps has gradually risen. The primary drawback of this flap is that the safe harvest range of this flap is restricted, and it can solely be employed to repair small- and medium- sized defects. Although numerous anatomical and clinical studies have been conducted on this flap, most of these studies concentrate on investigating the location, diameter, and pedicle length of the perforators of the medial sural artery (Banjongleelahong et al., 2024; Sampieri et al., 2024). There is a limited amount of literature reporting on the causes of partial necrosis of this flap. The aim of this study was to explore the causes underlying the limited boundary of the medial sural perforator flap via an anatomical study and to present the results of our clinical utilization of this flap for the reconstruction of infected defects.

## Methods

### Anatomical observation

Five fresh lower limb specimens were obtained from five patients (aged 25–47 years) in our department who had recurrent malignant bone tumors in the upper two - thirds of the femur and underwent amputation at the upper femur level (n = 2) or hip joint level (n = 3). Femoral artery catheterization was performed, and a gelatin–lead oxide mixture (gelatin: lead oxide: water = 5 g: 100 g: 100 mL) was injected. Then, the integuments were stripped and radiographed (Peng et al., 2022a; Tao et al., 2024; Zhou et al., 2021).

### Clinical application

#### Patients

We carried out a retrospective analysis of the data from 7 patients who underwent the reconstruction of infected soft - tissue defects in the regions of the knee, middle and distal leg, and heel using MSAP flaps in our department between April 2023 and July 2025. This study was conducted in accordance with the Declaration of Helsinki and received approval from the Ethics Committee of the Third Xiangya Hospital Central South University.

#### Surgical procedure

After the anesthesia is administered, the patient is placed in the supine position. Radical debridement of the recipient site is performed, and a paper template is cut according to the size of the wound for subsequent use.

The axis of the flap is defined as the line connecting the midpoint of the popliteal fossa to the posterior border of the medial malleolus. The key point is the site of the perforator of the medial sural artery, which is located using Doppler ultrasound preoperatively. If Doppler ultrasound fails to locate the perforator, the key point can be designed at a distance of 8.0 cm and/or 12.0 cm from the popliteal fossa. Design the flap centered around the key points according to the above paper template. To avoid excessive tension after the flap covers the defect, the flap should be designed to be 1 cm larger than the defect.

The anterior border of the flap was first incised, and dissection was performed superficially on the deep fascia layer to explore the perforator. If necessary, adjust the design of the flap according to the location of the perforators. Make an incision in the deep fascia approximately 0.5 cm anterior to the perforator. Dissect in a retrograde fashion along the perforator until the medial sural artery is reached. Subsequently, proceed with the retrograde dissection along the medial sural vessels to an appropriate length. Then, create an incision along the posterior edge of the flap. Liberate the posterior edges of the perforator and the main trunk of the medial sural artery via the same approach, and fully harvest the flap. Finally, cover the recipient site using either the pedicled transfer or free graft method, and suture the donor site directly.

#### Postoperative management

The patient was advised to rest in bed, while the affected leg was maintained in an elevated position. The low molecular weight heparin (3200 IU, Q12h) was administered subcutaneously for anticoagulation, while papaverine (30 mg, Q8h) was given intramuscularly to prevent vasospasm. These procedures were continued until 7 days postoperatively. The antibiotic was administered to manage the infection based on the results of bacterial culture and drug susceptibility, as well as the consultation of the clinical pharmacist.

#### Evaluation parameters

The patient’s demographics (i.e., sex and age), etiologies, flap dimensions, reconstruction outcomes, infection control rate, recurrence rate, and follow - up results were collected to evaluate the effectiveness and reliability of the MSAP flaps for reconstructing the infected soft tissue defects.

## Results

### Anatomic observation

**Figure 1:**
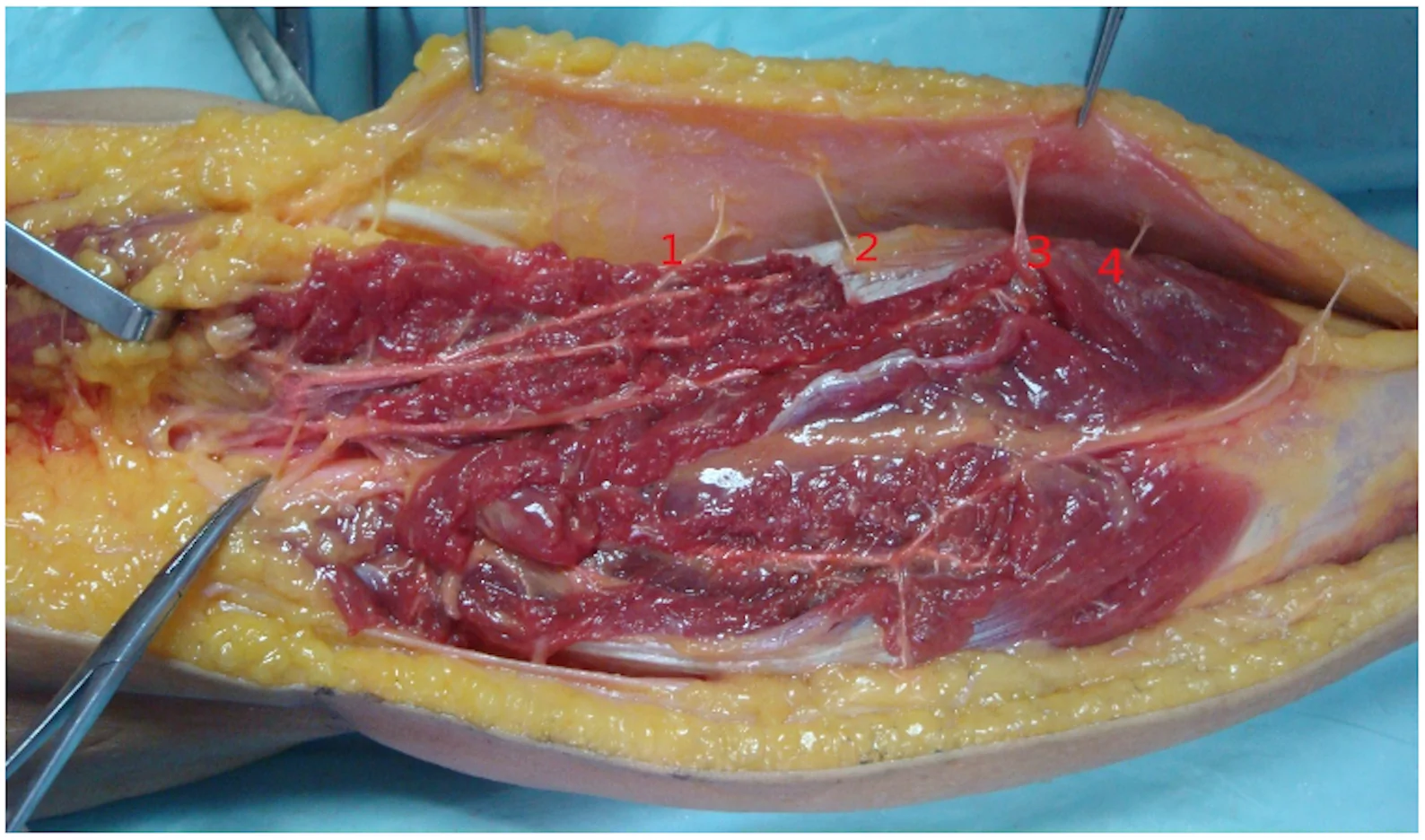
Anatomical observation of the medial sural artery and its perforators: In the image, 1, 2, 3, and 4 denote the perforators of the medial sural artery, corresponding to 3, 9, 10, and 11 in **Figure 2** respectively.

In all five cases, the medial sural arteries originated from the popliteal artery, having an origin diameter of 2.2 ± 0.2 mm. Along its course, the medial sural artery gave rise to a mean of 6 perforators (ranging from 4 to 8 perforators). These perforators were mainly distributed at a distance of 1.8 ~ 6.5 cm from the posterior midline and 6.6 ~ 17.0 cm from the popliteal crease. Among them, two perforators were relatively thick and could be clearly discerned with the naked eye. The distances of these two perforators from the popliteal crease were 7~10.5 cm and 13.5~15.5 cm respectively, and their mean diameters were 0.7 mm and 0.84 mm respectively. The diameters of the remaining perforators were comparatively small. (**Figure 1**).

**Figure 2:**
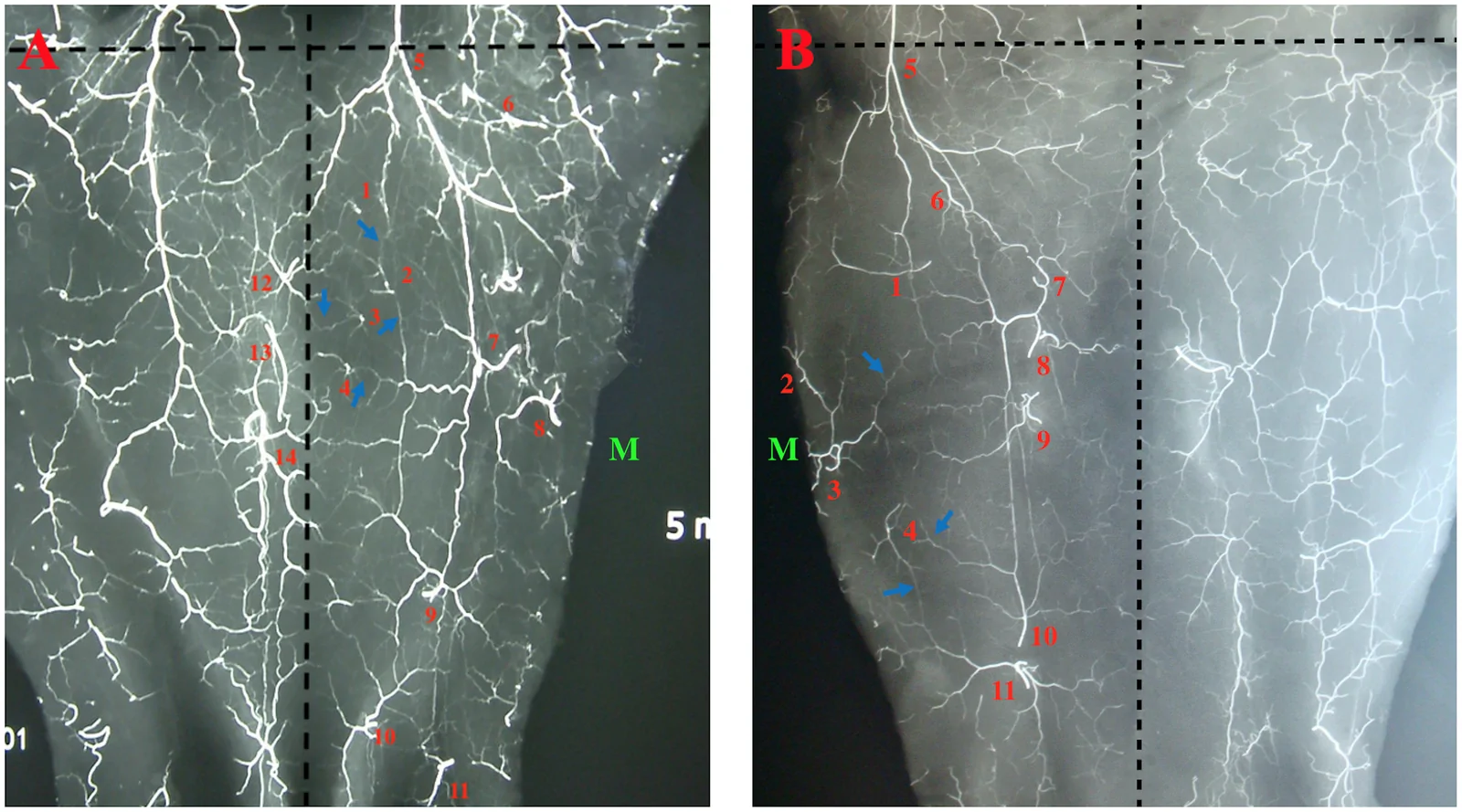
Radiograph showing arterial vasculature of leg integument [5, 6, 13]. **(A)** The integument was incised along intermalleolar line and anterior midline. Transverse and longitudinal dotted lines represent popliteal crease and posterior midline, respectively. **(B)** The integument was incised along intermalleolar line and posterior midline. Transverse and longitudinal dotted lines represent popliteal crease and anterior midline, respectively. Adjacent perforators of the medial sural artery (1, 2, 3, 4) are interconnected by choke vessels. The perforators of the medial sural artery (1, 2, 3, 4) form choke anastomoses anteriorly with the perforators of the posterior tibial artery (1, 8, 9, 10, 11), cross the posterior midline of the calf posteriorly, and laterally anastomose with the nutrient vessels of the sural nerve (14) and the musculocutaneous perforators of the lateral sural artery (12, 13) via choke vessels. The blue arrows indicate choke anastomoses. (5) Saphenous artery. (6) Medial inferior genicular artery.

The arteriography revealed that the anastomoses between adjacent medial sural artery perforators, as well as those between medial sural artery perforators and surrounding perforators, were choke anastomoses. There are dense choke anastomoses between the medial sural artery perforators and the posterior tibial artery perforators inferiorly; between the medial sural artery perforators and the fasciocutaneous perforators of the posterior tibial artery anteriorly; and between the medial sural artery perforators and the sural nutrient vessels, the musculocutaneous perforators of the lateral sural artery, and the lateral cutaneous artery of the popliteal fossa laterally across the posterior midline of the calf posteriorly (**Figure 2**).

### Clinical application

The patient and flap characteristics and the reconstruction outcomes are summarized in **Table 1**.

**Table 1:** The patient and flap characteristics and the reconstruction outcome.

No	Gender	Age	Defect sites	Causes	Defect size (cm)	Flap size (cm)	Outcome
1	Male	51	Knee	Trauma	6x4	7x4	survival
2	Female	37	middle leg	Trauma	9.5x5	10.5x6	survival
3	Male	55	Lower leg	Chronic ulcer	10x4	11x5	survival
4	Male	57	middle leg	Trauma	9×4	10×5	survival
5	Male	53	Lower leg	Trauma	9×4	10×5	survival
6	Female	66	Heel	Trauma	11×4	12×5	survival
7	Male	43	middle leg	Trauma	13×5	14×6	survival

Among the 7 patients, 5 were males and 2 were females, with an average age of 51.7 years (range: 37 - 66). The etiologies of the defects were trauma-related events in 6 patients and chronic osteomyelitis with chronic ulcer in 1 patient. All the defects were associated with various degrees of infection, and none of the defects were caused by diabetes mellitus or peripheral arterial disease. The size of the defects ranged from 6 cm × 4 cm to 13 cm × 5 cm.

All of the 7 flaps survived completely without any indications of arterial insufficiency or venous congestion. The wounds in the donor areas healed successfully in one stage, and no recurrent infection or other complications were encountered.

**Figure 3:**
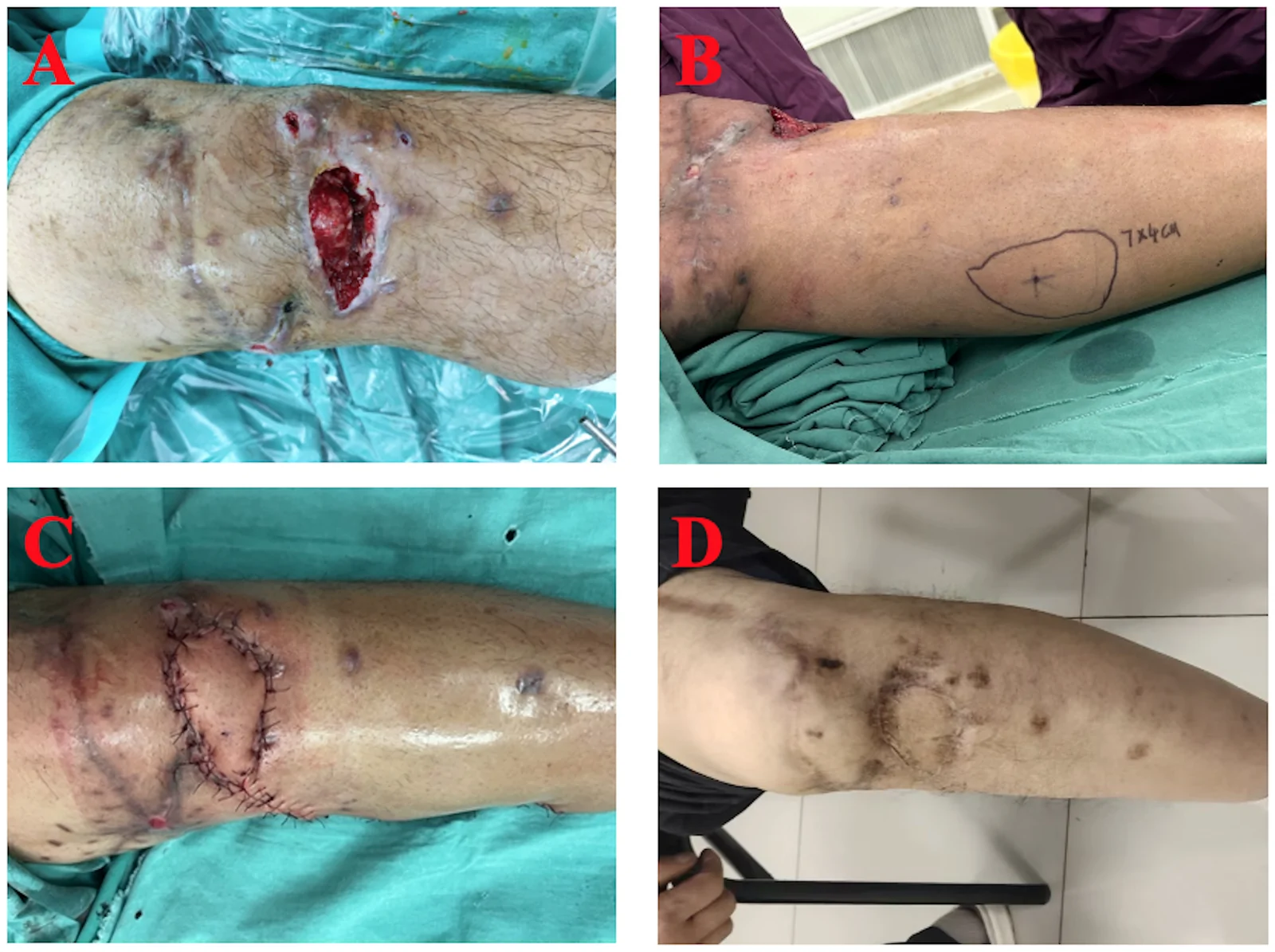
A 51-year-old male (Patient 1 in **Table 1**) presented with an infected soft-tissue defect on the anterior aspect of the knee that resulted from a car accident. **(A)**. Following aggressive debridement, a pedicled medial sural perforator flap with dimensions of 7 cm × 4 cm was designed on the same leg **(B)**. The flap was transferred to reconstruct the defect **(C)**, and the donor site was directly sutured. At a 33 - month follow - up, the flap survived completely, and there was no relapse of infection **(D)**.

**Figure 4:**
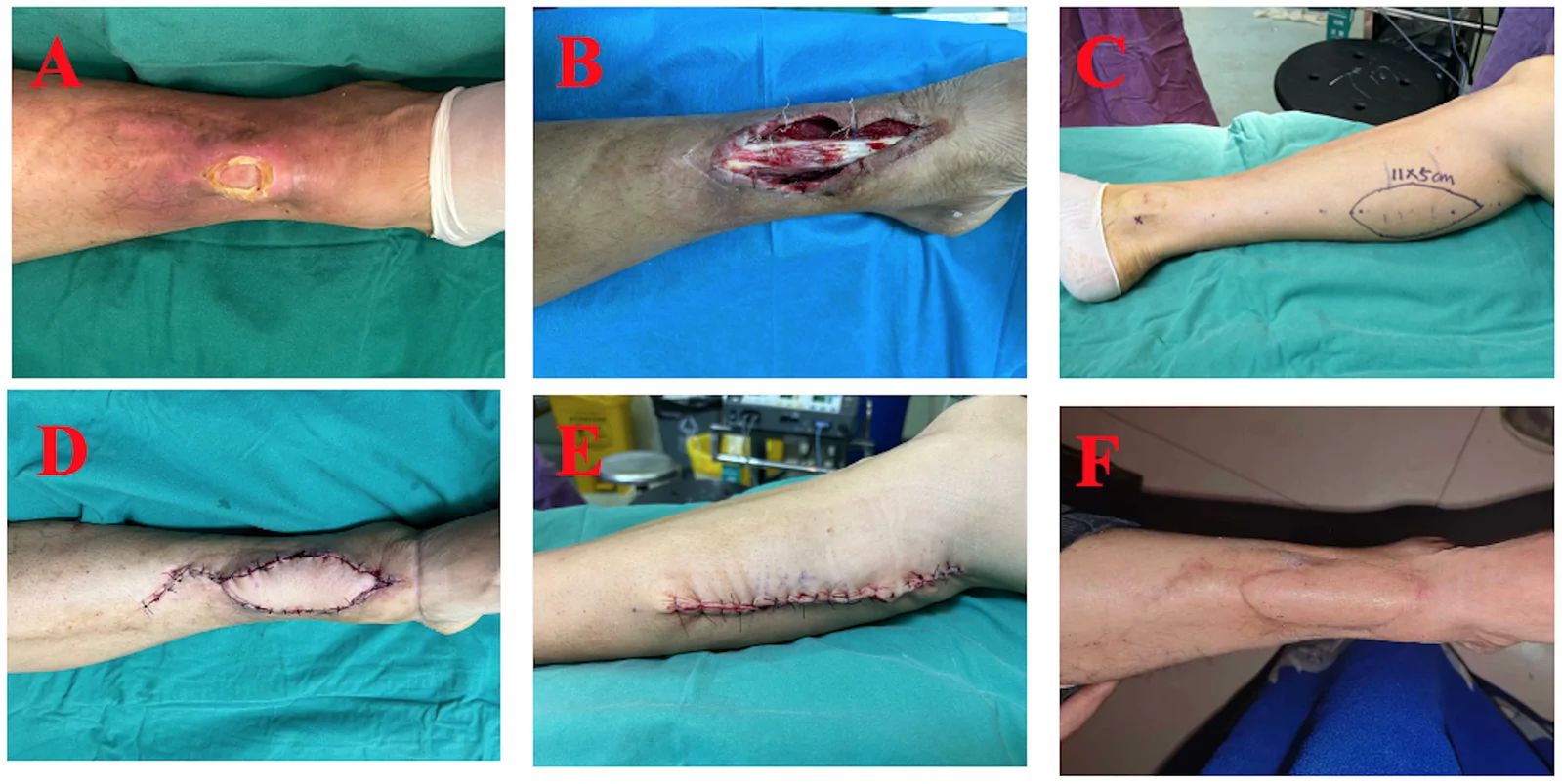
A 55-year-old male (Patient 3 in **Table 1**) presented with tibial osteomyelitis and a chronic ulcer in the distal part of the left lower leg **(A)**. After aggressive lesion debridement **(B)**, a medial sural perforator flap was designed on the right lower leg, and the dimensions of the flap were 11 cm × 5 cm **(C)**. The flap was utilized to reconstruct the defect of the left lower leg **(D)**, and the donor site was directly sutured **(E)**. At a 6 - month follow - up, the flap survived completely, and osteomyelitis was controlled. The patient was satisfied with the aesthetic appearance and the reconstructed outcome **(F)**.

The follow-up procedures were performed in all 7 patients with a period ranging from 6 to 33 months (mean of 11.8 months). No clinical signs of persistent or recurrent infection, wound dehiscence, or flap necrosis were detected during the available follow - up period. All the patients regained stable ambulation without the assistance of the crutch or orthosis and return their daily life. None of the patients complained about the aesthetic appearance and reconstructed outcome. Typical cases are presented in **Figures 3** and **4**.

## Discussion

The MSAP flap is widely used clinically for soft tissue defect repair and reconstruction due to its numerous advantages, including a consistent anatomy of the perforators, abundant blood supply, and reliable survival (Dattilo et al., 2025; Madonna et al., 2024). When this flap is utilized to repair defects in the distal part of the calf and the ankle area, the texture of the flap closely resembles that of the adjacent tissues (Jandali et al., 2018; Lin et al., 2021). Furthermore, the flap features a moderate thickness and is non-bulky, leading to excellent aesthetic outcomes for the reconstruction of the recipient area. Moreover, the flap can be harvested without incorporating the gastrocnemius muscle and deep fascia, enabling direct closure of the donor site with relatively minor damage (Al-Himdani et al., 2020). This method adheres to the principle currently advocated by the world’s microsurgical community for defect reconstruction, which emphasizes better reconstruction of the recipient area and less damage to the donor site.

The necrosis of the flap was the main complication of the flap transplantation, and the main cause was the insufficient blood supply of the flap (Peng et al., 2022b, Peng et al., 2022c). Literature reports indicate that when adjacent perforasomes are connected by vessels with an undiminished diameter (true anastomosis), flaps can be dissected over a relatively large area spanning multiple perforasomes (Peng et al., 2025; Zhou et al., 2021). When adjacent perforasomes are connected by vessels with a gradual decrease in diameter (choke anastomoses), the harvestable range of the flap will be significantly reduced (Z. Cao et al., 2025; Ji et al., 2024). Numerous studies indicate that when the multiterritory perforator flap is connected by choke vessels, the perforators can nourish their respective perforasomes (corresponding to the anatomical site) and can also traverse one choke anastomosis to nourish the adjacent perforasomes (corresponding to the hemodynamic site). Partial necrosis of the flap frequently occurs in the area beyond the second choke anastomosis (corresponding to the potential site) (Callegari et al., 1992; Ji et al., 2024). Numerous clinical and anatomical studies have suggested that the harvest range of the MSAP flap is restricted, and it can only be employed to repair small- to medium- sized defects (Jandali et al., 2018; Zhang et al., 2025). Nevertheless, the relevant causes and influencing factors have not been precisely determined. This study, based on anatomical research, ascertained that the diameters of the perforators of the medial sural artery are predominantly small. Additionally, choke anastomoses are present both among the perforators of the medial sural artery and between the perforators of the medial sural artery and those of the surrounding source arteries. Therefore, when a flap with a comparatively large area is harvested across multiple perforasomes, the risk of partial flap necrosis will increase substantially.

Early coverage of the defects and sufficient blood supply are the guarantees for the repair of infected defects. Defect coverage can mitigate the invasion of exogenous bacteria and the loss of tissue fluid and nutrients. Adequate blood supply transports antibiotics and anti-infective factors to the infected area and eliminates infected and necrotic tissues from the body via blood circulation. The MSAP flap is characterized by a rich blood supply. It is capable of covering the defect while simultaneously providing sufficient blood supply to the recipient area. Consequently, it represents an effective approach for repairing infected defects. In the case of a defect associated with chronic osteomyelitis, a chimeric perforator flap incorporating a gastrocnemius muscle flap can be designed (S. Cao et al., 2025). The muscle flap is employed to fill the cavity, and the skin flap is utilized to cover the wound, which is more favorable for the control of infection. In this study, all 7 cases presented with infected defects, and all flaps achieved complete survival. The infections were effectively managed, the fractures healed primarily, and all patients resumed normal life and work. Moreover, no recurrence of infection - related complications was observed during the follow-up period. The patients were satisfied with both the aesthetic and functional outcomes.

The present study has certain limitations. The anatomical research only observed factors influencing the harvestable dimension of the MSAP flap and did not establish a safe maximum dimension for the MSAP flap. The sample size of anatomical study and clinical application cases were relatively small. This study was a single - center, retrospective study. It lacked a comparator group, and it was unable to establish causality or determine a safe maximum flap size of the MSAP flap. The specimens were obtained from patients undergoing amputation for malignant tumors, which may lead to potential selection bias. Additionally, the limited direct correlation between anatomical findings and clinical outcomes may be a limitation of this study. Future studies with larger sample sizes and multicenter collaborations can further investigate the vascular characteristics and reliability of MSAP flap.

## Conclusion

The MSAP flap can be effectively utilized to repair small- and medium- sized infected defects on the extremities. The possible factors influencing the limited harvestable boundary of this flap are that all adjacent perforators are anastomosed through choke vessels.

## Data Availability

The raw data supporting the conclusions of this article will be made available by the authors, without undue reservation.
